# Peritonism

**Published:** 1900-11-24

**Authors:** 


### PERITONISM.

Writing on the subject of acute intestinal obstruction, Dr. Ernest Maylard[1] insists that the aim should be not so much to arrive at a precise diagnosis as to the cause of the condition met with, as to decide whether or not the case is one for operation. This is the really important point in regard to treatment, and he holds that, without troubling about causes, it is possible to distinguish a condition which, however produced, gives a positive indication as to treatment. To this condition Gruber has applied the term "peritonism," and when it is present the abdomen must be opened. No cases present greater difficulties to the practitioner in their early stage than those in which the patient is suddenly seized with acute pain in the abdomen, a pain which temporarily prostrates and seems to partially paralyse the nervous and vascular systems.

When confronted with such a case the first question is as to the gravity or otherwise of the symptoms. Are they indicative of some gross lesion or are they not?

In deciding this it may be said that if the symptoms be taken collectively and in association with the acute seizure of pain, it will be found as a rule that severe colic, renal, biliary, or intestinal, does not proportionately affect either the vascular or the nervous system; in other words, that the pulse is not markedly increased in rapidity or lessened in power; nor are the peripheral vessels contracted so as to produce any striking pallor or shrunken aspect of the features. Vomiting, which may have occurred at first, is not continuous, and the symptoms are seen, in the course of an hour or so, to diminish rather than increase. On the other hand, when the case is a grave one, it will be found, as a rule, that, accompanying the excruciating seizure of pain, the pulse is likely to be increased in rapidity and small, the face pinched and pale, the eye listless, the forehead bathed in perspiration, and the features generally indicative of suffering. The temperature may be anything between subnormal and a degree or two above normal. The surface of the body may seem chilled, the abdomen may be rigid and painful at certain places, and vomiting is usually present. These are the initial symptoms of a grave attack, and although time, no doubt, would clear up the nature of the case, time cannot be afforded.

The point is, that we have here a group of symptoms which, whatever their cause, demand operation. It is not suggested that operation may not be demanded by other conditions also, but that when we find ourselves face to face with this state of "peritonism," we need not go farther—we need not trouble about minutiæ of diagnosis, operation is required, and in operating we shall find out the rest.

[1] Practitioner, Nov.

